# Review of the Chinese species of the genus *Reptalus* Emeljanov, 1971 (Hemiptera, Fulgoromorpha, Cixiidae), with description of a new species

**DOI:** 10.3897/zookeys.1290.186750

**Published:** 2026-08-25

**Authors:** Sha-Sha Lv, Zi-Zhong Li, Yan Zhi, Yu-Bo Zhang, Xiang-Sheng Chen, Lin Yang

**Affiliations:** 1 Guizhou Key Laboratory of Agricultural Biosecurity, Guizhou University, Guiyang, Guizhou, 550025, China Guizhou Key Laboratory of Agricultural Biosecurity, Guizhou University Guiyang China; 2 Institute of Entomology, Guizhou University, Guiyang, Guizhou, 550025, China Institute of Entomology, Guizhou University Guiyang China; 3 The Provincial Special Key Laboratory for Development and Utilization of Insect Resources of Guizhou, Guizhou University, Guiyang, Guizhou, 550025, China The Provincial Special Key Laboratory for Development and Utilization of Insect Resources of Guizhou, Guizhou University Guiyang China; 4 Laboratory Animal Center, Guizhou Medical University, Guiyang, Guizhou, 550025, China Laboratory Animal Center, Guizhou Medical University Guiyang China; 5 Anshun University, College of Agriculture, Anshun, Guizhou, 561000, China Anshun University, College of Agriculture Anshun China

**Keywords:** Checklist, cixiids, distribution, identification key, Oriental region, planthopper, taxonomy

## Abstract

The Chinese species of the genus *Reptalus* Emeljanov, 1971 (Hemiptera, Fulgoromorpha, Cixiidae) are reviewed. A new species, *R. inaequalis* Lv, Li & Chen, **sp. nov**., from Fujian Province, China, is described and illustrated. This new species belongs to the subgenus *Pererepa*. The female genitalia of two species within this genus, R. (R.) arcbogdulus (Dlabola, 1965) and R. (P.) quadricinctus (Matsumura, 1914), are described and illustrated herein for the first time. A checklist and an updated identification key to the Chinese species of the genus are provided.

## Introduction

The cixiid planthopper genus *Reptalus* Emeljanov, 1971 (Hemiptera, Fulgoromorpha, Cixiidae, Cixiinae, Pentastirini, Pentastirina) was established by Emeljanov (1971), with *Cixius quinquecostatus* Dufour, 1833 designated as its type species. Subsequently, Emeljanov (2020) transferred the type fixation to *Flata artemisiae* Becker, 1865, based on the findings of Webb et al. (2013), a circumstance that was also discussed by Tishechkin and Emeljanov (2024). The genus currently comprises four subgenera—*Reptalus* Emeljanov, 1971, *Trepalus* Emeljanov, 1995, *Palvanus* Emeljanov, 2015, *Pererepa* Emeljanov, 2020—and 34 species distributed in Palaearctic, Nearctic and Oriental regions (Emeljanov 2020; Dmitriev et al. 2022; Bourgoin 2026). *Reptalus* is readily distinguished from the remaining Pentastirina by the following combination of characters: vertex extremely broad and rectangular; pygofer asymmetric; anal segment with caudal margin broadly and irregularly deeply concave; apex of gonostyli with a typical, large, introrse process on inner side; aedeagus with right-basal periandrial position giving rise to a long process, often accompanied by a small process at its base (Bai et al. 2015).

Chinese records of *Reptalus* were summarized by Guo and Wang (2007), who listed two species: R. (P.) quadricinctus (Matsumura, 1914) and R. (P.) basiprocessus Guo & Wang, 2007. Bai et al. (2015) added R. (P.) shunxiwuensis Bai, Guo & Feng, 2015, raising the total to three species. Luo et al. (2022) further increased the Chinese fauna to six species in their nationwide census of Cixiidae.

In the present contribution, the Chinese species of *Reptalus* are reviewed, and a new species, R. (P.) inaequalis sp. nov., from Fujian Province, China, is described and illustrated in detail. Additionally, the female genitalia of two species are described and illustrated for the first time. Consequently, the known diversity of the genus in China is elevated to seven species. A checklist and an updated identification key to all Chinese species are provided.

## Materials and methods

Specimens included in the present study were collected using a sweeping net. Type specimens and other examined materials are deposited in the Institute of Entomology, Guizhou University, Guiyang, Guizhou Province, China (**IEGU**).

Morphological terminology follows Bourgoin (1987) for male genitalia, Bourgoin (1993) and Chen and Zhi (2023) for female genitalia, and Bourgoin et al. (2015) for wing venation. Descriptions and illustrations were based on dry-preserved specimens. All measurements are given in millimeters (mm). External morphology and drawings were done under a Leica MZ 12.5 stereomicroscope. Photographs were taken using KEYENCE VHX-6000 and VHX-1000 systems. The photographs and illustrations were subsequently processed and assembled into plates using Adobe Photoshop 6.0.

## Taxonomy

### Reptalus

Taxon classificationAnimaliaHemipteraCixiidae

Emeljanov, 1971

DF9979D8-B466-5107-9D96-8BBEBB6A665D

Reptalus Emeljanov, 1971: 621.

#### Type species.

*Flata artemisiae* Becker, 1865, by subsequent designation in Emeljanov 2020: 198.

#### Diagnosis.

For the diagnosis of *Reptalus* see Bai et al. (2015: 32).

#### Distribution.

Palaearctic, Nearctic and Oriental regions.

##### Checklist of Chinese species of *Reptalus* Emeljanov, 1971

R. (R.) arcbogdulus (Dlabola, 1985); China (Beijing, Shanxi), Mongolia.

R. (P.) basiprocessus Guo & Wang, 2007; China (Fujian, Guizhou, Hebei, Hubei, Hunan, Jiangsu, Qinghai, Sichuan, Zhejiang).

R. (P.) iguchii (Matsumura, 1914); China (Beijing, Guizhou, Hunan), South Korea, Japan.

R. (P.) inaequalis sp. nov.; China (Fujian).

R. (P.) quadricinctus (Matsumura, 1914); China (Anhui, Beijing, Fujian, Guizhou, Hebei, Henan, Hubei, Hunan, Jiangsu, Jilin, Shaanxi, Sichuan, Shanghai, Zhejiang), Japan, Russia, South Korea.

R. (R.) quinquecostatus (Dufour, 1833); China (Chongqing), Armenia, Austria, Azerbaijan, Belgium, Bulgaria, Czech Republic, France, Georgia, Germany, Greece, Hungary, Iran, Italy, Kazakhstan, Korea, Kosovo, Kyrgyzstan, Luxembourg, Malta, Moldova, Poland, Palestine, Portugal, Romania, Russia, Serbia, Slovakia, Slovenia, Spain, Switzerland, Tadzhikistan, Tajikistan, Türkiye, Ukraine, United Kingdom.

R. (P.) shunxiwuensis Bai, Guo & Feng, 2015; China (Anhui, Sichuan, Zhejiang).

##### Key to Chinese species of *Reptalus* Emeljanov, 1971

**Table:** 

1	Periandrium without dorsal tooth	**Reptalus (Reptalus) 2**
–	Periandrium with dorsal tooth	**Reptalus (Pererepa) 3**
2	Periandrium with a very long and curved process at right basal area; endosoma with three spinous processes (Emeljanov 2015: fig. 142 2)	**R. (R.) arcbogdulus (Dlabola, 1985)**
–	Periandrium without a very long and curved process at right basal area; endosoma with two spinous processes (Emeljanov 2015: fig. 143 2)	**R. (R.) quinquecostatus (Dufour, 1833)**
3	Forewing with two brown transverse bands (Bai et al. 2015: fig. 1)	**R. (P.) quadricinctus (Matsumura, 1914)**
–	Forewing without such bands	**4**
4	Periandrium (Fig. 2K–N) with forked process at dorsal part, ventral part with a hawk-beaked process	**R. (P.) inaequalis Lv, Li & Chen, sp. nov**.
–	Periandrium without such processes at dorsal and ventral parts	**5**
5	Basiventral area of endosoma giving rise to a small spine (Guo and Wang 2007: fig. 2)	**R. (P.) basiprocessus Guo & Wang, 2007**
–	Basiventral area of endosoma without spine	**6**
6	Periandrium with five processes, the length of short process at right basal area approximately one-fifth of long one (Bai et al. 2015: figs 32, 33)	**R. (P.) shunxiwuensis Bai, Guo & Feng, 2015**
–	Periandrium with three processes, the length of short process at right basal area approximately one-third of long one (Rahman et al. 2012: fig. 1H)	**R. (P.) iguchii (Matsumura, 1914)**

### Reptalus (Reptalus) arcbogdulus

Taxon classificationAnimaliaHemipteraCixiidae

(Dlabola, 1985)

83CD8F96-2070-551B-B7EC-23591D8E5CA3

Fig. 1

Oliarus arcbogdulus Dlabola, 1965: 87.
Reptalus arcbogdulus (Dlabola, 1965): Emeljanov 1971: 622, 1982: 111.
Reptalus (Proreptalus) arcbogdulus (Dlabola, 1965): Emeljanov 2015: 209.
Reptalus (Reptalus) arcbogdulus (Dlabola, 1965): Emeljanov 2020: 198.

#### Material examined.

China • 2♂♂2♀♀: Shanxi Province, Hengqu County, Lishan National Nature Reserve; 35°39'N, 111°94'E; sweeping, 25 July 2012; Pei Zhang leg.; IEGU.

#### Diagnostic characters.

Total length: male 5.0–5.7 mm, female 5.7–6.9 mm. Forewings without brown marks. Chaetotaxy of hind tarsomere 7/7, second tarsomere of hind tarsi bearing five platellae. Pygofer nearly symmetrical, posterior margin with an angular process medially. Gonostyli asymmetrical. Periandrium of aedeagus without dorsal tooth, with a very long and curved process at right basal area; endosoma with three slender spinous processes.

#### Supplementary description.

**Figure 1.**
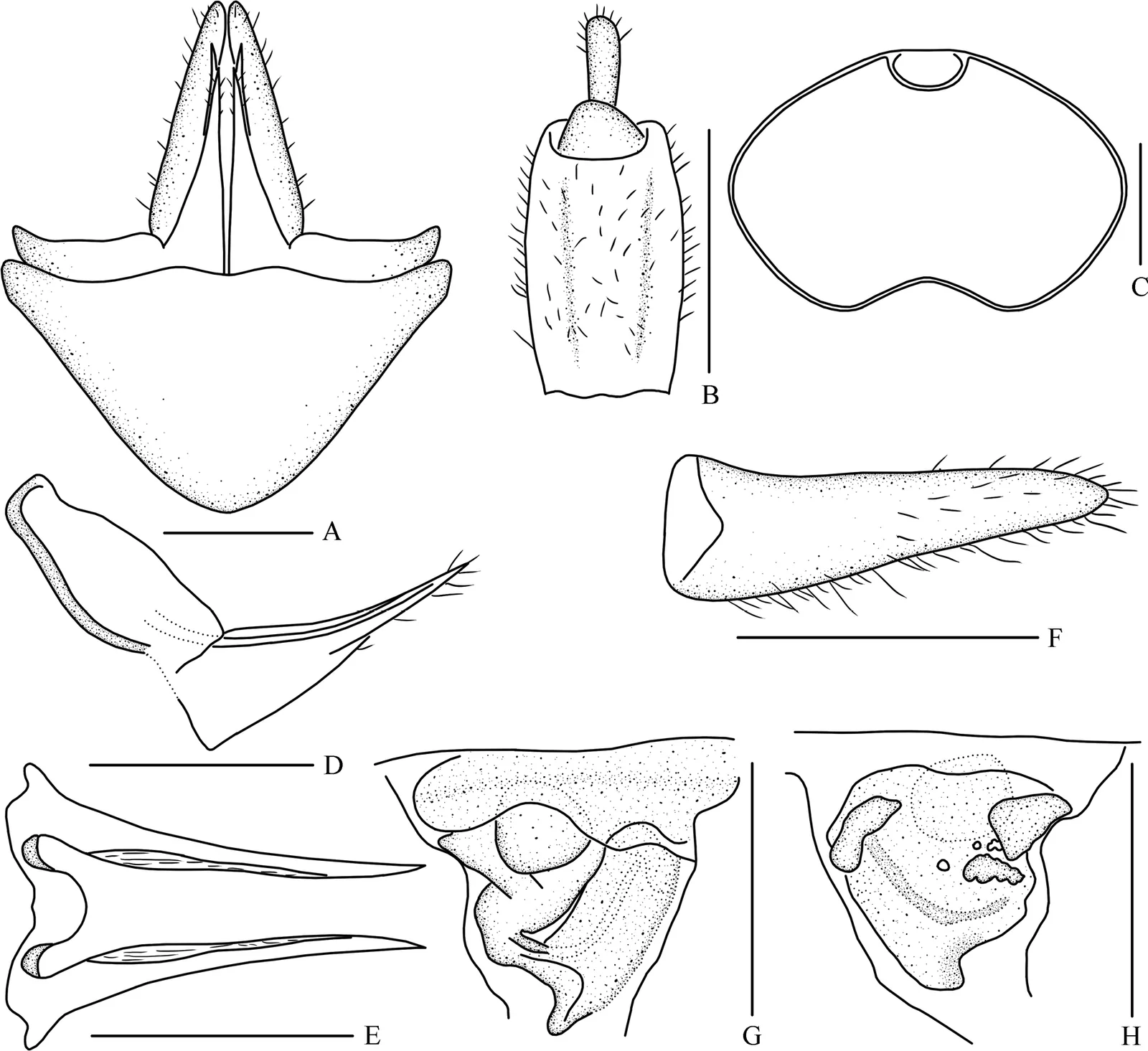
Reptalus (Reptalus) arcbogdulus (Dlabola, 1985), female. **A**. Genitalia, ventral view; **B**. Anal segment, dorsal view; **C**. Pygofer, caudal view; **D**. Gonapophysis VIII and gonocoxa VIII, lateral view; **E**. Gonapophysis IX, lateral view; **F**. Gonoplac, inner lateral view; **G**. Posterior vagina, ventral view; **H**. Posterior vagina, dorsal view. Scale bars: 0.5 mm (**A–H**).

***Female genitalia***. Sternite VII (Fig. 1A) in ventral view with anterior margin arcuate, shorter than wide (1:1.90). Pygofer (Fig. 1C) subelliptical, 1.52 times as long as wide in caudal view, dorsal and ventral margins recessed arcuately. Anal segment (Fig. 1B) subrectangular, 1.73 times as long as wide in dorsal view, anal style fingerlike. Gonapophysis VIII (Fig. 1D) narrowly triangular. Gonapophysis IX (Fig. 1E) slender, slightly curved, tapering to apex. Gonoplac (Fig. 1F) clavate, 3.01 times as long as wide in lateral view. Posterior vagina pattern as shown in Fig. 1G, H.

#### Distribution.

China (Beijing, Shanxi), Mongolia.

### Reptalus (Pererepa) basiprocessus

Taxon classificationAnimaliaHemipteraCixiidae

Guo & Wang, 2007

EEACAC9E-D5B2-5171-8204-F9BD6C048D24

Reptalus basiprocessus Guo & Wang, 2007: 276; Bai et al. 2015: 37.
Reptalus (Pererepa) basiprocessus Guo & Wang, 2007: Emeljanov 2020: 199.

#### Material examined.

No specimen examined.

#### Diagnostic characters.

Total length: male 6.3–7.0 mm, female 7.0–8.0 mm. Forewings without brown marks, whitish-hyaline. Chaetotaxy of hind tarsomere 7/7, second tarsomere of hind tarsi bearing five platellae. Pygofer asymmetrical, right side with a triangular lobe; left side sinuated, slightly convex in middle. Gonostyli greatly asymmetrical. Periandrium of aedeagus with five processes, with dorsal tooth, right basal area giving rise to two processes, longer one sickle-like; endosoma with three spinous processes, two slender processes along median posterior margin, basiventral area giving rise to a small spine.

#### Distribution.

China (Fujian, Guizhou, Hebei, Hubei, Hunan, Jiangsu, Qinghai, Sichuan, Zhejiang).

### Reptalus (Pererepa) iguchii

Taxon classificationAnimaliaHemipteraCixiidae

(Matsumura, 1914)

6554515C-5819-5123-9827-713AE85D3FC5

Oliarus iguchii Matsumura, 1914: 419.
Reptalus (Reptalus) iguchii (Matsumura, 1914): Rahman et al. 2012: 38.
Reptalus iguchii (Matsumura, 1914): Hayashi and Fujinuma 2016: 326.
Reptalus (Pererepa) iguchii (Matsumura, 1914): Emeljanov 2020: 199.

#### Material examined.

No specimen examined.

#### Diagnostic characters.

Total length: male 6.2–7.0 mm, female 7.9–8.2 mm. Forewings of female with scattered dark brown spots on near middle forming a transverse band, and apical half with some scattered dark brown spots, male without above color pattern. Chaetotaxy of hind tarsomere 7/7, second tarsomere of hind tarsi bearing five platellae. Pygofer with asymmetrical laterodorsal processes. Gonostyli asymmetric, length of right apical process one-third of left one. Periandrium of aedeagus with three processes, endosoma with two slender processes.

#### Distribution.

China (Beijing, Guizhou, Hunan), Japan, South Korea.

### Reptalus (Pererepa) inaequalis

Taxon classificationAnimaliaHemipteraCixiidae

Lv, Li & Chen sp. nov.

E4D02403-DA7C-575A-80CC-D5978E460951

https://zoobank.org/F55EE616-8640-4F2C-99A4-D631D5BBE2A7

Figs 2, 3

#### Diagnosis.

**Figure 2.**
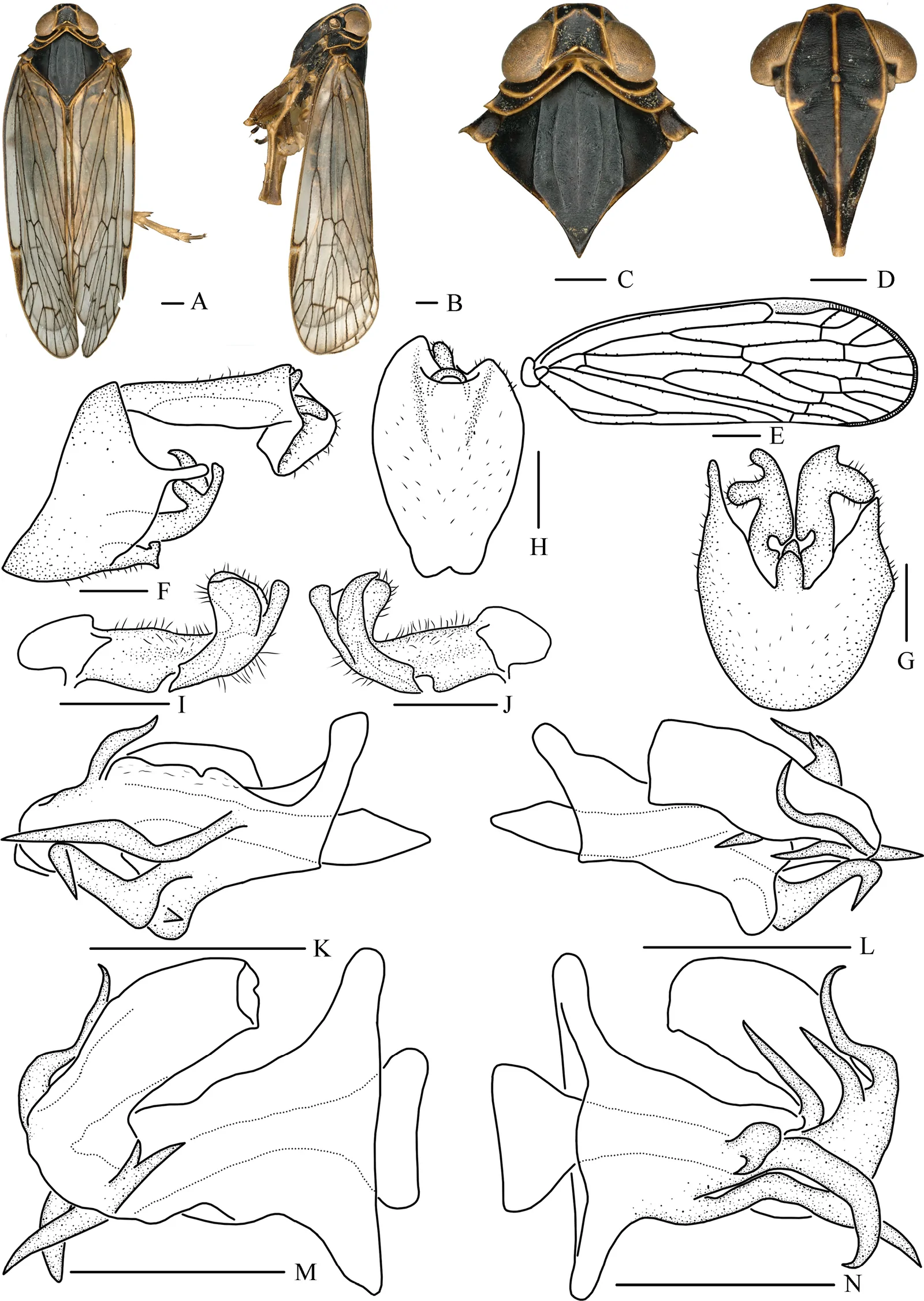
Reptalus (Pererepa) inaequalis Lv, Li & Chen, sp. nov., male. **A**. Habitus, dorsal view; **B**. Habitus, lateral view; **C**. Head and thorax, dorsal view; **D**. Frons, ventral view; **E**. Forewing; **F**. Genitalia, lateral view; **G**. Pygofer and gonostyli, ventral view; **H**. Anal segment, dorsal view; **I**. Right gonostyli, inner lateral view; **J**. Left gonostyli, inner lateral view; **K**. Aedeagus, right side; **L**. Aedeagus, left side; **M**. Aedeagus, dorsal view; **N**. Aedeagus, ventral view. Scale bars: 0.5 mm (**A–D, F–N**); 1.0 mm (**E**).

The salient features of the new species include: total length (Fig. 2A, B) of male 8.5–9.5 mm, female 9.3–10.4 mm; chaetotaxy of hind tarsomere 8/9–10, second tarsomere of hind tarsi bearing seven platellae; pygofer (Fig. 2F, G) asymmetrical, fingerlike process at middle part of left lobe; anal segment (Fig. 2F, H) and gonostyli (Fig. 2I, J) asymmetrical; Aedeagus (Fig. 2K–N) in total with seven processes, short process of periandrium in ventral side hawk-beaked, dorsal side with a forked process, unequal in length.

**Figure 3.**
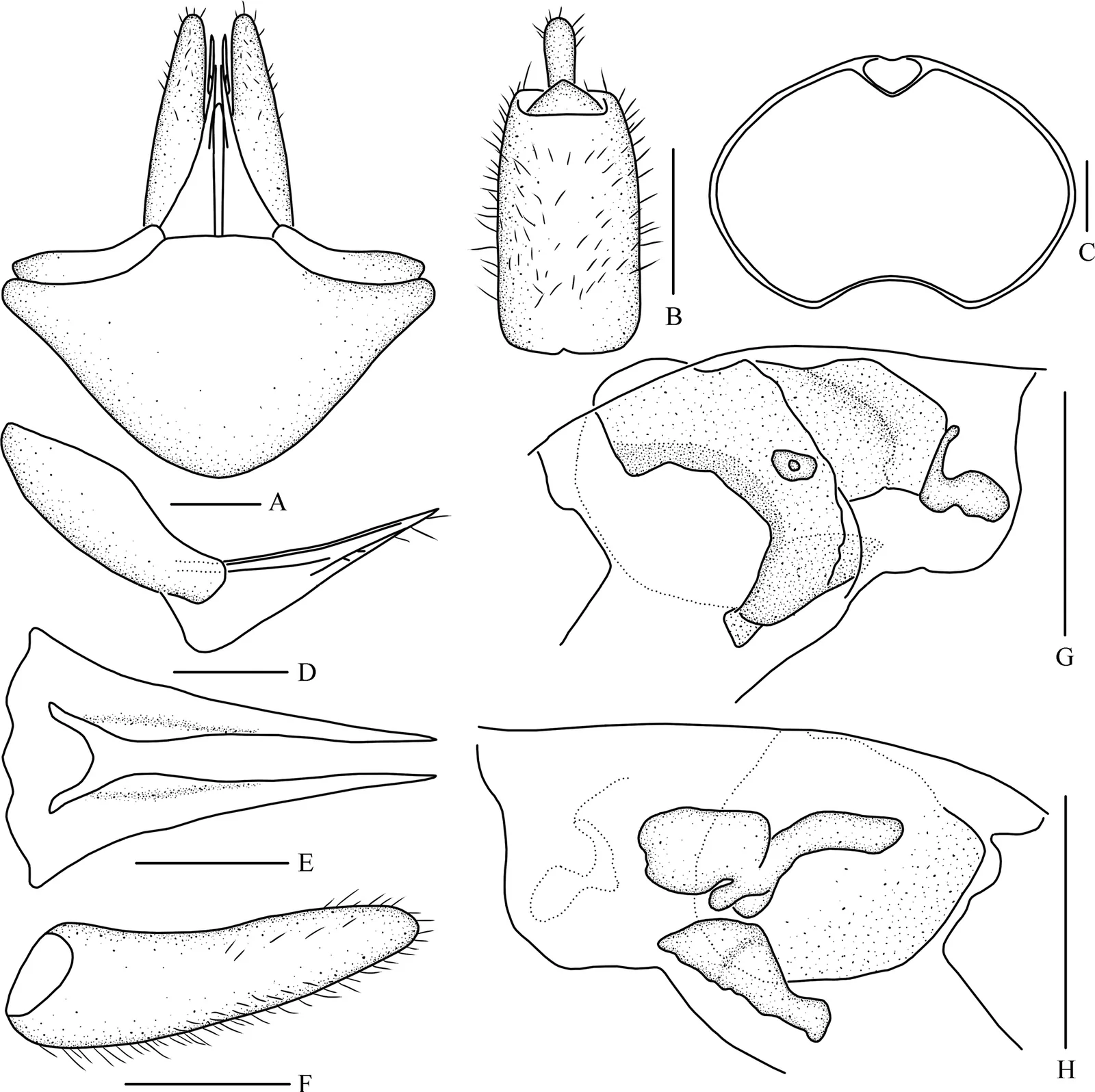
Reptalus (Pererepa) inaequalis Lv, Li & Chen, sp. nov., female. **A**. Genitalia, ventral view; **B**. Anal segment, dorsal view; **C**. Pygofer, caudal view; **D**. Gonapophysis VIII and gonocoxa VIII, lateral view; **E**. Gonapophysis IX, lateral view; **F**. Gonoplac, inner lateral view; **G**. Posterior vagina, ventral view; **H**. Posterior vagina, dorsal view. Scale bars: 0.5 mm (**A–H**).

#### Type material.

***Holotype***: China • ♂; Fujian Province, Shaowu City; 27°36'N, 117°49'E; sweeping, 28 May 2012; Jian-Kun Long leg.; IEGU. ***Paratypes***: China • 4♂♂, 8♀♀; Fujian Province, Shaowu City; 27°36'N, 117°49'E; sweeping, 28 May 2012; Jian-Kun Long, Hu Li, Zhi-Min Chang and Wei-Cheng Yang leg.; IEGU.

#### Description.

***Measurements***. Total length: male 8.5–9.5 mm (*N* = 5), female 9.3–10.4 mm (*N* = 8).

***Coloration***. Body (Fig. 2A, B) black. Carinae of vertex, frons and pronotum yellowish brown. Eyes brown. Ocelli yellowish brown, semi-translucent. Rostrum yellowish brown, apical part dark brown. Lateral margins of mesonotum yellowish brown to brown. Tegula blackish brown, outer part yellowish brown. Forewings semi-translucent, light grayish-brown, stigma dark brown, veins and tubercles brown, as shown in Fig. 2E.

***Head and thorax***. Vertex (Fig. 2A, C) 1.55 times as long as wide, width at apex narrower than at base (1:1.43), anterior margin slightly curved and convex, subapical carina and posterior margin angular recessed, lateral carina developed, median carina indistinct. Frons (Fig. 2D) shorter in middle line than wide at widest portion (about 1:1.32), widest at nearly apex, lateral carina developed, median longitudinal carina prominent, percurrent, forked at apex. Median ocellus distinct. Clypeus (Fig. 2D) with median carina. Pronotum (Fig. 2A, C) short, with an indistinct medial carina and two distinct postocular carinae, posteriorly deeply and wide-angulately emarginated in medial area, curving laterally, collar-like. Mesonotum (Fig. 2A, C) longer than 2.74 times pronotum and vertex combined, with five longitudinal carinae. Forewings (Fig. 2E) 3.07 times as long as wide, with eleven apical cells and six subapical cells, ScP+RP forked slightly distad of fork CuA1+CuA2, r-m crossvein basad of fork MA+MP, Pcu and A1 united centre of clavus. RP 2 branches, MA apically trifurcated, MP apically bifurcated. Hind tibia with three lateral spines, chaetotaxy of hind tarsomere 8/9–10, second tarsomere of hind tarsi bearing 7 platellae.

***Male genitalia***. Pygofer (Fig. 2F, G) asymmetrical, in lateral view, fingerlike process at middle part of left lobe extended caudally, ventral and dorsal margins of medioventral process formed a small process apically; in ventral view, dorsal margin nearly U-shaped concavely, medioventral process columnar. Anal segment (Fig. 2F, H) with two sides extended laterally, broad, with two processes at apex, right side larger than left side; in lateral view, dorsal margin nearly straight, ventral margin gradually widens towards the end; in dorsal view, 1.62 times as long as wide. Anal style fingerlike, beyond anal segment. Gonostyli (Fig. 2F, G, I, J) asymmetrical, three lamellar processes at apical part, curved inward; in ventral view, apical part bifurcated, irregular in shape; in inner lateral view, ventral margin sunken at middle part, formed a lamellar process, left style arc-shaped apically, right style sharp-angled. Aedeagus (Fig. 2K–N) in total with seven processes. Periandrium in ventral margin with two spinous processes, short one hawk-beaked shaped, directed ventrocephalad, long one curved, basal 3/4 directed caudally, apical part directed ventrocephalad; dorsal margin with a forked process, unequal in length, directed dorsocephalad; middle part with a long spinous process, curved, directed caudally. Endosoma curved cephalad, basal ventral margin with three spinous processes, middle one shortest, curved one longest, directed dorsocephalad.

***Female genitalia***. Sternite VII (Fig. 3A) in ventral view with anterior margin arcuate, flat at middle, shorter than wide (1:1.81). Pygofer (Fig. 3C) 1.80 times as long as wide in caudal view, dorsal and ventral margins recessed arcuately. Anal segment (Fig. 3B) subrectangular, 1.90 times as long as wide in dorsal view, anal style fingerlike. Gonapophysis VIII (Fig. 3D) narrowly triangular. Gonapophysis IX (Fig. 3E) slender, inner margin arcuately protruding at basal 1/3, tapering to apex. Gonoplac (Fig. 3F) clavate, 3.25 times as long as wide in lateral view. Posterior vagina pattern as shown in Fig. 3G, H.

#### Distribution.

China (Fujian).

#### Etymology.

The species name is derived from the Latin word “*inaequalis*”, referring to the dorsal margin of the periandrium with an unequally bifurcate spinose process apically.

#### Remarks.

This species is similar to R. (R.) arcbogdulus (Dlabola, 1985), but differs from the latter in: (1) right-basal spinose process of periandrium arcuate, not encircling toward the left (right-basal spinose process of periandrium arcuately encircles toward the left in R. (R.) arcbogdulus); (2) dorsal margin of periandrium with an unequally bifurcate spinose process apically (dorsal margin of periandrium without an unequally bifurcate spinose process apically in R. (R.) arcbogdulus); and (3) periandrium with two spinose processes in ventral margin (periandrium without two spinose processes in ventral margin in R. (R.) arcbogdulus).

### Reptalus (Pererepa) quadricinctus

Taxon classificationAnimaliaHemipteraCixiidae

(Matsumura, 1914)

4E51B6ED-9F27-5331-AEDF-2B3E96F36240

Fig. 4

Oliarus quadricinctus Matsumura, 1914: 419.
Oliarus bizonatus Kato, 1932: 228; Chou et al. 1985: 20.
Oliarus trifasciatus Metcalf, 1954: 605; Van Stalle 1991: 17.
Reptalus quadricinctus (Matsumura, 1914): Emeljanov 1971: 622; Anufriev and Emeljanov 1988: 464; Liang 2005: 429; Guo and Wang 2007: 27.
Reptalus (Reptalus) quadricinctus (Matsumura, 1914): Rahman et al. 2012: 35; Bai et al. 2015: 35; Emeljanov 2015: 215, 2020: 199; Hayashi and Fujinuma 2016: 326.

#### Material examined.

China • 9♂♂8♀♀: Guizhou Province, Sinan County, Qinggangpo Town; 27°88'N, 108°01'E; sweeping, 2 August 2016; Yong-Shun Ding and Liang-Jing Yang leg.; IEGU. 9♂♂9♀♀: Guizhou Province, Libo County, Wengang Town; 25°25'N, 107°91'E; sweeping, 4 July 2010; Xiao-Hui Hou, Pei Zhang and Yan-Li Zheng leg.; IEGU. 1♂: Henan Province, Dengfeng City, Shaolin Temple; 34°51'N, 112°95'E; sweeping, 17 July 2002; Xiang-Sheng Chen leg.; IEGU. 4♂♂1♀: Hebei Province, Zunhua City, Liupanying Village; 40°18'N, 117°69'E; sweeping, 5 August 1999; Wen-Ming Cui leg.; IEGU. 4♂♂3♀♀: Hubei Province, Dabieshan National Nature Reserve; 31°11'N, 115°57'E; sweeping, 2 July 2014; Zheng-Xiang Zhou and Mei-Na Guo leg.; IEGU. 3♂♂5♀♀: Shaanxi Province, Zhouzhi County, Houzhenzi Town; 33°86'N, 107°85'E; sweeping, 7 August 2010; Pei Zhang leg.; IEGU.

#### Diagnostic characters.

Total length: male 5.0–5.3 mm, female 5.6–6.2 mm. Vertex about 0.7 times longer than broad. Forewings with four brown marks. Chaetotaxy of hind tarsomere 7/7, second tarsomere of hind tarsi bearing five platellae. Pygofer asymmetrical, right side with a triangular lobe, left side with two moderate-sized convexities. Aedeagus in total with seven processes; periandrium with five processes, right basal area giving rise to two processes, left ventroapical area with two moderate-sized processes, dorsal area with a medium-sized acuminate right apical process; endosoma with two slender processes along median posterior margin.

#### Supplementary description.

**Figure 4.**
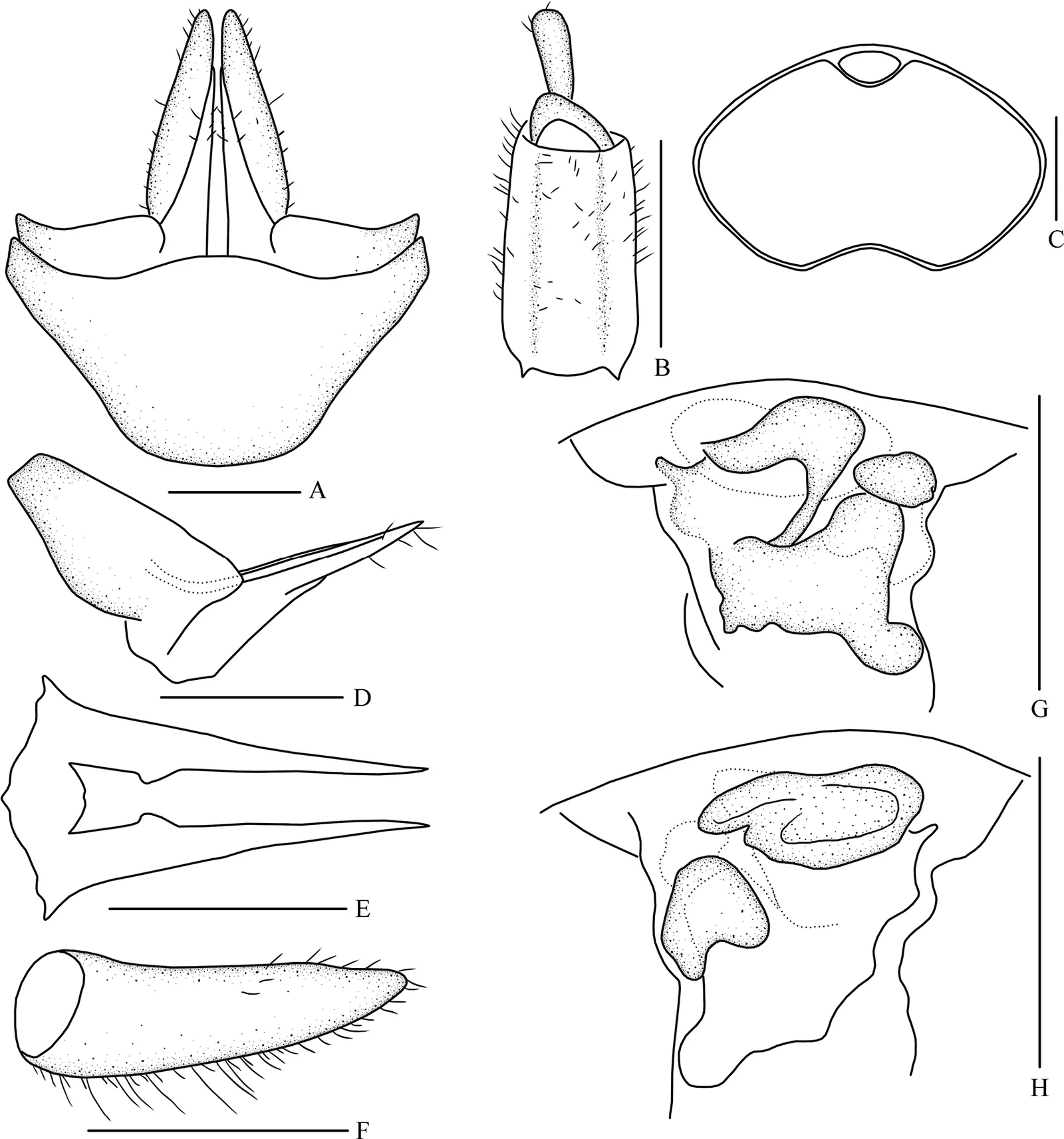
Reptalus (Pererepa) quadricinctus (Matsumura, 1914), female. **A**. Genitalia, ventral view; **B**. Anal segment, dorsal view; **C**. Pygofer, caudal view; **D**. Gonapophysis VIII and gonocoxa VIII, lateral view; **E**. Gonapophysis IX, lateral view; **F**. Gonoplac, inner lateral view; **G**. Posterior vagina, ventral view; **H**. Posterior vagina, dorsal view. Scale bars: 0.5 mm (**A–H**).

***Female genitalia***. Sternite VII (Fig. 4A) in ventral view with anterior margin arcuate, middle part convex arcuately, shorter than wide (1:2.02). Pygofer (Fig. 4C) 1.62 times as long as wide in caudal view, dorsal and ventral margins recessed arcuately. Anal segment (Fig. 4B) subrectangular, 1.92 times as long as wide in dorsal view, anal style fingerlike. Gonapophysis VIII (Fig. 4D) narrowly triangular. Gonapophysis IX (Fig. 4E) slender, inner margin with angular process at basal 1/3. Gonoplac (Fig. 4F) clavate, 3.13 times as long as wide in lateral view. Posterior vagina pattern as shown in Fig. 4G, H.

#### Distribution.

China (Anhui, Beijing, Fujian, Guizhou, Hebei, Henan, Hubei, Hunan, Jiangsu, Jilin, Shaanxi, Shanghai, Sichuan, Zhejiang), Japan, Russia, South Korea.

### Reptalus (Reptalus) quinquecostatus

Taxon classificationAnimaliaHemipteraCixiidae

(Dufour, 1833)

BD365ED4-03FE-51EC-92C7-E737347384EE

Cixius quinquecostatus Dufour, 1833: 224.
Oliarus quinquecostatus (Dufour, 1833): Fieber 1872: 3; Kusnezov 1937: 168.
Oliarus melanochaetus Fieber, 1872: Puton 1875: 113; Fieber 1876: 198; Ferrari 1882: 83; Lambertie 1904; Kusnezov 1928: 52; Metcalf 1936: 82; Nast 1972: 26; Galinichev 2008: 37; Webb et al. 2013: 62.
Reptalus melanochaetus (Fieber, 1876): Emeljanov 1971: 622.
Reptalus quinquecostatus (Dufour, 1833): Emeljanov 1971: 622; Lodos and Kalkandelen 1980: 23; Holzinger et al. 2003: 127; Jović et al. 2009: 1055, 2010: 238; Bertin et al. 2010: 3; Cvrković et al. 2010: 222, 2011: S130; Drobnjaković et al. 2010: 313; Mozaffarian and Wilson 2011: 14.
Reptalus (Reptalus) melanochaetus (Fieber, 1876): Galinichev 2014: 273.
Reptalus (Reptalus) quinquecostatus (Dufour, 1833): Galinichev 2014: 274; Mozaffarian 2018: 480; Emeljanov 2020: 198.
Reptalus (Proreptalus) melanochaetus (Fieber, 1876): Emeljanov 2015: 213.
Reptalus (Proreptalus) quinquecostatus (Dufour, 1833): Emeljanov 2015: 209.
Reptalus (Proreptalus) melanochaetus (Fieber, 1876): Emeljanov 2015: 213.

#### Material examined.

No specimen examined.

#### Diagnostic characters.

Total length: male 4.9–6.8 mm, female 5.5–8.3 mm. Vertex about 0.8 times longer than broad. Forewings without brown marks, whitish-hyaline. Chaetotaxy of hind tarsomere 7/7, second tarsomere of hind tarsi bearing five platellae. Pygofer nearly symmetrical, posterior margin curved. Gonostyli asymmetric, inner side with a slender spinous process. Periandrium of aedeagus with five processes, right side with three processes, one extremely narrow; middle part with apical rounded process, left side with relatively long process, wide at base, apical part bifurcated two small processes; endosoma with two processes, ventral one conical.

#### Distribution.

China (Chongqing), Armenia, Austria, Azerbaijan, Belgium, Bulgaria, Czech Republic, France, Georgia, Germany, Greece, Hungary, Iran, Italy, Kazakhstan, Korea, Kosovo, Kyrgyzstan, Luxembourg, Malta, Moldova, Poland, Palestine, Portugal, Romania, Russia, Serbia, Slovakia, Slovenia, Spain, Switzerland, Tadzhikistan, Tajikistan, Türkiye, Ukraine, United Kingdom.

### Reptalus (Pererepa) shunxiwuensis

Taxon classificationAnimaliaHemipteraCixiidae

Bai, Guo & Feng, 2015

3095D39F-E77A-53D0-A83E-D65351A1A81A

Reptalus shunxiwuensis Bai, Guo & Feng, 2015: 38.
Reptalus (Pererepa) shunxiwuensis Bai, Guo & Feng, 2015: Emeljanov 2020: 199.

#### Material examined.

No specimen examined.

#### Diagnostic characters.

Total length: male 5.8–6.0 mm. Vertex about 0.5 times longer than broad. Chaetotaxy of hind tarsomere 7/7, second tarsomere of hind tarsi bearing five platellae. Pygofer asymmetrical, right side with a triangular lobe; left side sinuated, slightly convex in middle. Gonostyli asymmetrical, left style with distal ramus acuminated, proximal ramus rounded at apex, inner introrse process forefinger-like; right style distally unequally bilobed, inner introrse process flattened, molar-like. Periandrium with five processes, right basal area giving rise to two processes, dorsal member long and large, sickle-like, ventral member smaller, apical two-thirds curved 90 degrees upward; dorsal side with a process; endosoma with two slender acuminate processes along median posterior margin.

#### Distribution.

China (Anhui, Sichuan, Zhejiang).

## Discussion

Zhi et al. (2017, 2018, 2019) examined both external and internal structures of the female genitalia in cixiids, demonstrating that characteristics of the posterior vaginal wall serve as a key diagnostic character for species-level identification within the genera *Neocarpia*, *Oecleopsis* and *Tsauria*. Rahman et al. (2012) reported pronounced sexual dimorphism in R. (P.) iguchii, which underscores the critical importance of female genitalic characters for the taxonomy within this genus. Given the scarcity of such data for *Reptalus*, the present study provides detailed illustrations and descriptions of the female genitalia for three species, establishing a reference framework for future systematic studies on the genus.

Based on published records and our field surveys, the genus currently comprises 35 valid species, which are distributed across the Oriental, Palaearctic and Nearctic Regions. Seven of these have been documented in China, occurring in the Northeast (NEC), North (NC), Qinghai–Tibet (QT), Southwest (SWC), Central (CC) and South (SC) zoogeographical subregions (Luo et al. 2022; Bourgoin 2026). *Reptalus* (*R.*) *quinquecostatus* (Dufour, 1833) exhibits the widest global distribution, whereas R. (P.) quadricinctus (Matsumura, 1914) is the most broadly distributed species within China. The present study extends the known distribution of the latter to include Henan and Hebei provinces, confirming its ongoing range expansion in China. Continued intensified sampling is expected to further extend its distribution and to reveal additional, possibly undescribed taxa.

Among the documented host associations of Cixiidae, and of planthoppers in general, species of *Reptalus* represent the most extensively recorded genus. To date, larvae or adults have been collected from representatives of 17 plant orders: Apiales, Asterales, Brassicales, Caryophyllales, Cornales, Fabales, Fagales, Geraniales, Hamamelidales, Lamiales, Malpighiales, Pinales, Poales, Ranunculales, Rosales, Sapindales and Solanales (Lodos and Kalkandelen 1980; Bertin et al. 2010; Lörinczi 2012; Rahman et al. 2012; Bourgoin 2026). During our field surveys, adults of R. (P.) inaequalis sp. nov. were collected from bamboo (Poaceae: Bambusoideae), indicating that bamboos may constitute an additional, previously unrecorded host resource for this species.

## Supplementary Material

XML Treatment for Reptalus

XML Treatment for Reptalus (Reptalus) arcbogdulus

XML Treatment for Reptalus (Pererepa) basiprocessus

XML Treatment for Reptalus (Pererepa) iguchii

XML Treatment for Reptalus (Pererepa) inaequalis

XML Treatment for Reptalus (Pererepa) quadricinctus

XML Treatment for Reptalus (Reptalus) quinquecostatus

XML Treatment for Reptalus (Pererepa) shunxiwuensis

## Additional information

### Artificial Intelligence (AI) use

The authors accept full responsibility for the content of the manuscript, including the disclosure of any use of AI.

No AI tools were used in the preparation of this manuscript.

### Author ORCIDs

S.-S. Lv https://orcid.org/0000-0001-5353-5082

Z.-Z. Li https://orcid.org/0000-0003-1609-9992

Y. Zhi https://orcid.org/0000-0003-1826-8139

Y.-B. Zhang https://orcid.org/0000-0002-6118-6190

X.-S. Chen https://orcid.org/0000-0001-9801-0343

L. Yang https://orcid.org/0000-0002-7841-5156

### Data availability

All of the data that support the findings of this study are available in the main text.
